# An exploratory study on the feasibility and appropriateness of family psychoeducation for postpartum women with psychosis in Uganda

**DOI:** 10.1186/1471-244X-13-131

**Published:** 2013-05-08

**Authors:** Janet Nakigudde, Anna Ehnvall, Florence Mirembe, Seggane Musisi, Eija Airaksinen

**Affiliations:** 1 Department of Psychiatry, Makerere University College of Health Sciences, Kampala, Uganda; 2 Department of Public Health Sciences, Division of Public Health Epidemiology, Karolinska Institute, Stockholm, Sweden; 3 Department of Obstetrics and Gynecology, Makerere University College of Health Sciences, Kampala, Uganda; 4 Department of Clinical Neuroscience, Gothenburg University and Psychiatric Out-patient Clinic, Varberg, Sweden

**Keywords:** Psychoeducation, Evidence-based practices, Culture, Cultural sensitivity, Cultural adaptation

## Abstract

**Background:**

We explored how family psychoeducation could be made culturally sensitive for postpartum mothers with psychotic illness in a Ugandan setting.

**Methods:**

A qualitative multi-method approach using an already existing family psychoeducation Tool Kit was adapted to incorporate lay perceptions related to psychotic illness in the postpartum period in this Ugandan setting. The participants consisted of postpartum women with psychotic illness, caregivers/family members, psychiatric nurses and psychologists. A modified version of a family psychoeducation programme for postpartum women with psychosis was formulated and pilot-tested.

**Results:**

Modifications in the standard family psychoeducation programme were both in the process and content of family psychoeducation. Under process, effective communication, cultural background, appropriate dress, involving only one family member, low literacy, and flexibility in timekeeping were raised. The theme of content yielded the incorporation of lay perceptions of mental illness, family planning, income generating, and an emphasis of premorbid and morbid personalities of the patients.

**Conclusion:**

The basic principles and assumptions underlying psychoeducation remained the same. Changes made in the process and content of family psychoeducation reflected the social, cultural and gender reality of the population.

## Background

Psychoeducation has been defined as the education of patients and their families that provides information, knowledge and the skills necessary for relapse prevention and health restoration of patients with psychiatric illness [1]. Psychoeducation is an evidence-based practice that leads to multiple positive health outcomes [2]. It has been reported to be an effective adjunctive psychosocial treatment for various mental health problems [3]. Randomized controlled trials have demonstrated increased efficacy of pharmacological intervention when combined with family psychoeducation in the treatment of mental health problems [4]. Although psychoeducation was initially developed to assist patients with schizophrenia, special modules have been developed for different psychological disorders such as bipolar disorder, eating disorders and personality disorders [2]. Family psychoeducation is an existing but not publicly available package especially in low income countries.

### Theoretical rationale, structure, and content of family psychoeducation

Mental health providers formulated family psychoeducation on the premise that indiscriminantly prescribing drugs would not cater for the needs of the patients and their caregivers. There was therefore a need to provide psychosocial interventions. Moreover these psychosocial interventions had to be well-structured. They realized the stressors that both the patients and their families were subjected to as a result of the patients not being understood and the inadequate information that the families were provided with, regarding mental illness. They therefore formulated a model of management that would direct clinicians, patients and families in positively influencing the course of pathophysiology in psychotic disorders. Family psychoeducation aids in the health providers’ partnering with family members of a patient afflicted with severe mental illness. This is for purposes of relapse prevention, fewer hospitalizations, decreased stigma, treatment compliance, a better involvement with family members and subsequently improved social support for the patient. Family psychoeducation aims at alleviating stress in both the patient and the family members, and it also provides greater knowledge regarding the nature of illness of a patient.

Various psychoeducation types have been described depending on group composition, which can be either single or multiple families. Single family psychoeducation involves one family in the education sessions whereas multi-family psychoeducation has multiple families that come together to enhance their knowledge on a mental illness that is affecting their relatives [5]. Psychoeducation that targets caregivers of psychiatric patients has resulted in similar outcomes to those for single or multi-family models of psychoeducation [6]. Multi-family psychoeducation comprises of 6 to 12 group members at a given time representing up to four families. The intervention can be short or long-term, depending on the frequency and duration of the sessions [7] which may be held in hospital or community settings. Psychoeducation sessions in clinical trials are reported to each last for about 60 minutes [8]. The process is highly structured with content aimed at enhancing knowledge, problem solving and skills training. The initial joining sessions, are used for participants to meet moderators individually. These are then followed by group sessions of education, problem solving and skills training [9].

Though universally acceptable, psychoeducation practice varies in different cultural settings. Psychoeducation of a modified culturally relevant intervention was carried out on Korean-American families for individuals with psychiatric illnesses [10]. The results showed improved coping skills, enhanced empowerment in dealing with crises, and a significant decrease in stigma. In China, a controlled study of a family psychoeducation intervention showed results of improved compliance, improved recognition of mental disturbances, and a better family/clinician partnership in the intervention group [3].

### Adaptation of psychological programmes

Various clinical trials have pointed to the effectiveness of culturally adapted psychological interventions. Theoretical frameworks have been hypothesized on the underlying assumptions of these cultural adaptations [11]. A Parent Child Interaction Therapy (PCIT) intervention was culturally adapted for Mexican American families, after identifying barriers to their accessing treatment. Focus groups and interviews of Mexican-American mothers, fathers, and therapists were used and modifications were drawn from which a culturally adapted version of PCIT was developed [12].

### Study setting

Uganda is a landlocked country found in East Africa with a total area of 240,000 square km. The country has a Gross National Income per citizen of US $ 490 (Worldbank database, 2011). It has a population of about 34,600,000 and a growth rate of 3.57% [13]. Culturally women in Uganda occupy a low social status and have a lower literacy rate of 56% compared to men with a literacy rate of about 71%. Uganda has a high fertility rate of 6.7 children per woman. The women of reproductive age constitute 23% of the whole population and on average women start having children at a young age. Twenty-five percent of all deliveries are by teenage girls. Ugandan women are exposed to high risks of death as a result of pregnancy complications. The lifetime risk of a Ugandan woman dying due to maternal causes is 1 in every 12. It has been estimated that for every Ugandan woman who dies as a result of a pregnancy- related cause, 20 to 30 women will become affected by short-term or long-term disabilities due to pregnancy or birth complications. Perinatal and maternal conditions have been ranked highest at 20% among the major causes of mortality and morbidity in Uganda [14].

### Cultural beliefs about psychiatric illness and postpartum psychosis in the Ganda culture in Uganda

The Ganda people of central Uganda culturally ascribe mental illness causation to supernatural forces [15,16]. They believe in highly organized systems of ancestral spirits, the top of which is *Lubaale.* Each family lineage or clan has various ancestral spirits representing dead ancestors called *mizimu*. *Lubaale* is a group of ancestral spirits from the same lineage. It is culturally believed that when appeased, then it can be protective but when begrudged, *Lubaale* can be vindictive and can supposedly cause illness which is then called a Kiganda illness [17]. This often includes severe mental illness manifesting as psychosis. It is commonly believed that this type of illness (psychosis) can only be treated by traditional African healers [17]. Postpartum psychotic illness in the Ganda culture of Uganda has also been culturally blamed on supernatural spirits supposedly brought on by the promiscuity of the woman during pregnancy [18]. In the Ganda culture, postpartum psychosis is therefore an illness laden with cultural family dynamics.

To a lesser extent postpartum psychosis has been attributed to the promiscuity of the male partner of the woman [19]. When the postpartum woman is aware that she has not been promiscuous, then she becomes suspicious of her male partner because of the cultural beliefs. She then feels a sense of betrayal by her partner but the partner is also warranted to feel betrayed by the postpartum woman. Therefore the cultural beliefs regarding postpartum psychosis place an emotional burden on postpartum women and their caregivers at a time when they are already distressed by the psychotic illness.

### Current services for women with postpartum psychotic illness in Uganda

The national mental referral hospital of Butabika provides space where a postpartum woman can be admitted into hospital with her infant and caregiver. This practice allows the postpartum woman to continue breastfeeding and emotionally bond with the infant while in hospital. Postpartum women receive the usual care of medication when they are taken to hospital. There is not much in the way of counseling or psychoeducation and the myths about postpartum psychosis are not demystified when postpartum women with their caregivers are in hospital. This creates a gap in the care for the women. To our knowledge, no research has been carried out specifically on postpartum women with psychotic illness to make the family psychoeducation programme culturally appropriate and gender sensitive. In this study we explored how family psychoeducation could be made culturally sensitive, feasible and appropriate for postpartum women with psychotic illness in Central Uganda.

## Methods

A qualitative multi-method design approach was employed using data from individual interviews, consultative meetings, group discussions, and observations from a piloted psychoeducation intervention for postpartum women with a current psychotic episode and their caregivers. The study was carried out at Butabika national mental referral and teaching hospital in central Uganda. The hospital catchment’s area is predominantly occupied by people of the Ganda tribe of central Uganda.

### Methods of recruiting different participant groups

#### Postpartum women and their caregivers

The postpartum women were consecutively recruited from the patients that had been admitted to the mental health facility on the female admission ward, with various forms of postpartum mental illnesses from December, 2009 to March, 2010. Their names were entered into a registry at the time when they were admitted and after they received their usual care of medication. At the time of discharge from the health facility, the study was introduced consecutively to these postpartum women and they were asked to voluntarily participate. Selection criteria for the women included the following: that the postpartum women presented with severe mental illness, had given birth in the past six months, had available caregivers, could communicate in the local language, and resided within a radius of 30 miles from the hospital facility. The respective caregivers that had provided the most social support during the time of acute illness were pointed out by the women and they too were asked to voluntarily participate in the study after an appropriate explanation. This was done with the permission of the postpartum women. Initially eight women and eight respective caregivers were recruited for this study. However two women with their caregivers dropped out; the first because they could not be traced using the telephone contact they had given to the study coordinator and the second because she had suffered HIV/AIDS complications and was sent to the village by her relatives. Consequently, six postpartum women and six respective caregivers attended the psychoeducation sessions and completed the study.

#### Psychiatric nurses participants’ method of recruitment

Five psychiatric nurses were included because they closely worked with women patients in the hospital. Four were selected because they were working on the female admission ward from which the postpartum women were recruited. One of the four nurses was the incharge of the female admission’s ward. The fifth nurse was recruited because she was incharge of the family planning unit of the mental hospital and constantly worked with women with mental health problems who needed family planning.

#### Clinical psychologists

These were recruited on the basis of a special interest in women’s mental health and they were purposively selected.

### Materials and data sources

#### A family psychoeducation implementation workbook as a data source

The family psychoeducation adapted in this particular study is an evidence-based practice that was originally developed for the Substance Abuse and Mental Health Services Administration (SAMHSA) in the US, based on evidence and expert opinions [2]. The family psychoeducation implementation workbook is part of the family psychoeducation tool kit. The workbook describes the rationale for carrying out psychoeducation, the principles of working with families, the core components and process of psychoeducation. The workbook describes the structure and content for the different family psychoeducation types for different mental disorders. It has a layout of how standard family psychoeducation should be carried out. The workbook was used to introduce the concept of family psychoeducation to the psychologists and psychiatric nurses. They were asked to read the workbook before the interviews were carried out.

Multiple data sources were used for the feasibility and appropriateness study. Family psychoeducation is an evidence based intervention with an approved structure, content and process (2). Failing to base our study on an already approved protocol would mean that time would be spent in reinventing what was already in place elsewhere and hence this was the reason for using it as a data source.

### A semi-structured interview and group guide

A semi-structured interview guide concerning family psychoeducation as described in the tool kit was formulated for the psychiatric nurses and psychologists. A semi-structured guide for the postpartum women and their caregivers was also formulated. The interviews lasted for approximately one and half hours on average.

#### Data management and analysis

Individual interviews with psychologists and psychiatric nurses were tape recorded and transcribed. The piloted family psychoeducation program involving postpartum women and their caregivers was also tape recorded and transcribed. From the transcribed data, we derived themes. These themes were then discussed in the consultative meetings to ensure that they were a true reflection of what the participants perceived culturally sensitive family psychoeducation would be. The psychologists and nurses participated in the first consultative meeting. However in the second meeting the psychologists, nurses, caregivers and the postpartum women all participated in the last consultative meeting. Both the women and their caregivers in the last consultative meeting were interviewed in the same group. Together they had participated and contributed to discussions in the psychoeducation and a harmonious relationship had been established.

There are two main reasons why the sessions in the administration of the twelve-week programme were tape recorded and transcribed. First, tape recording was done in order to facilitate the supervision of the 2 psychologists that were under supervision by a senior psychologist on a weekly basis. The tape recorded data aided in the psychologists’ understanding of their facilitating skills. The transcribed data from the administration of the twelve-week programme was used in order to validate the women’s and caregivers concerns and perceptions and this was one way by which themes of the results section were obtained.

#### Family psychoeducation for postpartum women

The first time that changes in the programme were made was after the psychologists’ and nurses’ interview. The family psychoeducation programme was then piloted on 12 participants. It was a group intervention. After the piloting of the modified programme, a discussion was held with the participants, the psychologists that had facilitated the program and the nurses and further changes were made to the already modified program. Psychiatric nurses were not directly involved in the facilitating of the program. The modified family psychoeducation program was moderated/facilitated by 2 psychologists at the same time and hence issues of inter-rater reliability did not arise. Their role was to facilitate the discussions in the psychoeducation and provide information to the postpartum women and their caregivers. The 2 psychologists had been trained and continued to be supervised throughout the duration of the program by a senior psychologist with a special interest in family psychoeducation.

#### Ensuring credibility of the final version of the modified family psychoeducation program

During the study, we closely followed the components that had been agreed upon for making up effective family psychoeducation in the US [9]. The data collectors/moderators were psychologists with a minimum qualification of a Masters degree in Clinical Psychology. The psychologists were members of the Uganda Counseling Association and the Uganda Clinical Psychology Association. All participants were fluent in Luganda; the local language in which the study was carried out. To determine the fidelity of the program, we used a check list of the family psychoeducation fidelity scale to ensure that the process and content of psychoeducation were adhered to [2]. Finally there was weekly supervision of the psychologists that pilot-tested the modified family psychoeducation program by the Principal Investigator (JN).

#### Ethical considerations and procedures

Permission to carry out the study was obtained from the School of Medicine’s Research and Ethics Committee of Makerere University, from the research committee of Butabika national mental referral and teaching hospital and lastly from the Uganda National Council of Science and Technology. Permission was also sought from the In-charges of the two hospital wards from which the mothers were recruited. Mothers that agreed to participate gave consent for their involvement in the study and consent indicating that they had agreed to their caregivers’ involvement in the psychoeducation programme. The caregivers in turn gave informed consent for their involvement in the family psychoeducation adaptation study. Finally, voluntary informed consent was obtained from the nurses and the psychologists.

Literature on psychoeducation models applicable to postpartum women indicates that the majority of women with postpartum psychotic illness have a DSM-IV diagnosis for bipolar disorder [20]. It was therefore agreed that a bipolar disorder psychoeducation model would be adapted for the women in the study. Firstly the psychiatric nurses and psychologists were individually approached and introduced to the basic principles of family psychoeducation using the workbook. They were requested to read specific chapters which included topics on the rationale for carrying out psychoeducation, the process of carrying out psychoeducation; joining, the education workshop, problem solving and other psychoeducation clinical models. This was followed by individual interviews with each of the nurses and the psychologists who were asked about the process and content of the program. After the interviews, the first consultative meeting was carried out with the nurses and psychologists. This meeting resulted in the first modifications in the process in the standard psychoeducation toolkit for bipolar disorder to make it more culturally sensitive.

Postpartum women along with their caregivers were then taken through this modified family psychoeducation program that included twelve sessions in twelve consecutive weeks. This was the pilot. The psychoeducation schedule was begun with both the women and the caregivers being invited for two individual joining sessions each. The woman had 2 sessions where she was not in the company of her caregiver and the caregiver also had 2 sessions where she or he was not in the company of the woman. This was followed by one conjoint session in which the postpartum woman and her caregiver were put in one session together and prepared for the subsequent nine sessions that would include other women and caregivers in the multi-family psychoeducation group. At the end of the 12 weeks the women and the caregivers together with the nurses and the psychologists had a second consultative meeting. Further changes in the content of standard family psychoeducation were incorporated into the psychoeducation programme.

## Results

### Socio-demographic characteristics of the participants

The study participants consisted of postpartum women, their caregivers, and healthcare providers that were Western trained. A total of 23 participants were recruited for the study. There were 5 psychiatric nurses, 2 psychologists, 8 postpartum women, and 8 caregivers. The sample size was small because we were looking for the smallest number of participants that would meet our criteria for pre-testing, modifying and adapting the psychoeducation programme. First it was important to bring all stakeholders together and these were the health providers that were taking care of the postpartum women with mental illness and secondly we included the postpartum women and their caregivers as the psychoeducation was aimed at them.

The psychiatric nurses and psychologists’ mean age was 40.4 and the range was between 26 and 48 years. The postpartum women’s mean age was 29 years with a range of 20 to 43 years. The mean age for the caregivers was 31 years with a range of 20 to 46 years. The majority of the women with their caregivers had attained education of between 1 and 7 years. Only one postpartum woman reported being married and all postpartum women presented with a bipolar disorder diagnosis as shown in Table 1.

**Table 1:** Socio-demographic characteristics of mothers and caregivers

		**Mothers**	**Caregivers**
**Age**	Oldest	43	46
	Youngest	20	20
	Mean age	29	31
**Sex**			
	Male	-	2
	Female	6	4
**Education**	1-7	3	2
	8-11 years	3	3
	>11 years	-	1
**Marital status**	widowed	2	-
	separated	2	1
	Single	-	1
	Visiting union	1	-
	Married	1	4
**Relationship to mother**	Parent	-	2
	Sibling	-	1
	Uncle	-	-
	Husband	-	2
	Friend	-	1
**Diagnosis**	Bipolar in manic phase	4	-
	Bipolar in depressive phase	2	-
	Organic psychosis	-	-

Results were obtained from three data sources; the individual interviews, the consultative group meetings, and the piloted family psychoeducation. Data collected at the different stages were merged to derive the themes presented in the result section. Individual interviews were carried out with the nurses and the psychologists. The individual interviews were done for purposes of eliciting information on how psychoeducation would be carried out in a most appropriate manner using the psychoeducation tool kit that the nurses and psychologists had read prior to the interviews. Many of the quotations below are from data obtained from the individual interviews. Data that arose from the individual interviews was used in modifying psychoeducation. The data from the consultative group meetings were obtained and merged with data obtained from individual interviews for a number of reasons. The first reason was for validating information obtained from the individual interviews. The second reason was for purposes of complementing data obtained from individual interviews. Some of the quotations in the results section are from the consultative group meetings. Data obtained from the consultative group meetings were used to modify the psychoeducation after the piloting had been carried out. The piloted psychoeducation programme enabled us to pick out issues that postpartum women and their caregivers emphasized. These issues were later raised in the consultative meetings and were incorporated in the already piloted psychoeducation programme.

These data sources yielded two broad themes:

i. The process of family psychoeducation

ii. The content of family psychoeducation

### Theme I: Process of family psychoeducation

The sub-themes within the process of family psychoeducation were: effective communication including the use of metaphors, cultural background including same language and same sex moderators, appropriate dress code, using one family member for the intervention, low literacy, and finally, flexibility in timekeeping.

a) Effective communication

Regardless of cultural diversity, psychological programs require that moderators or therapists that facilitate the psychological sessions acquire the basic knowledge and skills that are necessary for any therapeutic encounter [21]. To this effect both the nurses and psychologists stressed the importance of having moderators that were good in communication and counselling.

“Moderators should be able to effectively communicate with the mothers and they should be trained in counselling”

Psychiatric Nurse 1

The importance of effective communication was enhanced by use of locally appreciated idioms, metaphors and proverbs. It was apparent that the use of culturally accepted idioms aided in communicating with the women especially when there was need to make emphasis. One such proverb was *“Lubaale mbeera nga nembiro kwootadde”*. Translated literally, this proverb means *“God helps those who help themselves”*. The relevance of this metaphor/proverb to the mothers was that even when they still believed in their ancestors` powers to heal them, it was crucial that they help themselves by seeking and complying with Western treatment.

b) Cultural background including language, age and same sex moderators

Cultural background and language were discussed within two areas. One was that moderators or psychoeducation facilitators should use the same language as the postpartum women. This has been pointed out to be an enhancer in communication to clients [22] as it fosters effective communication between postpartum women, caregivers and moderators/facilitators.

“Psychoeducation facilitators should preferably be female, know the language the mothers know best and share the same cultural background with the postpartum women”

Psychiatric Nurse 3

It was deemed important that the facilitators of the psychoeducation process should be mature women that the mothers could easily relate with. To this effect one participant pointed out that:

“A moderator or psychoeducation facilitator should appear old enough to understand issues related to childbirth and marriage. Having a moderator who is too young will give the postpartum women the impression that she is not well versed with their problems related to childbearing”.

Psychiatric Nurse 4

Along with other issues, moderators or pyschoeducational facilitators brought into the sessions their own cultural beliefs, values and practices. If these were shared with the patients, then moderators stood better chances of understanding their clients and vice versa [23].

The other aspect of language was to tailor the psychoeducation discussion to the lay understanding of medical terms. It is very easy for professionals to be carried away in their explanations and use complicated terms that participants do not understand. Therefore, emphasizing the use of simple language that everybody would be comfortable with during the discussion was emphasized.

“There is a need to be cautious when in discussion because medical people are fond of using medical jargon that the rest of the people don’t understand. Sometimes when people don’t understand they may be afraid to ask, become bored and do not come back.”

Psychiatric Nurse 2

c) Appropriate dress code

There was a need to remain respectful of participants and this was highlighted by appropriate dress code by moderators or psychoeducational facilitators and respect for the diverse religious beliefs. For example, women psychologists needed to dress in long enough skirts or dresses that were considered culturally appropriate. Also, religious beliefs of the participants needed to be respected by making sure that participants were not judged according to their religious beliefs.

“If we are looking at making postpartum women and their caregivers comfortable in the discussions, then we should dress appropriately. Not exactly the way they dress but so that they stay respectful of us”.

“If we don’t show respect to their relatives, they will not want to come back. Whatever faith one belongs to, needs to be acknowledged with respect.”

Psychologist 1

d) Incorporating one family member

The nurses stressed the importance of working out a practical and sustainable program that was feasible when it came to implementation, especially in view of the limited resources. It was suggested that the program should initially incorporate one family member as opposed to many family members because it would be cheaper to obtain transport for one person than for the whole family. It would also mean that the rest of the family would be able to continue with their usual work as one respondent indicated below:

“It is not practical to have all family members attend a psychoeducation session in our setting. How many members should we allow, considering that we have extended families? It would be too expensive and not practical for all family members to attend. Moreover, their work would come to a standstill and the drop- out rate would be high”.

Participant 1 from one of the consultative meetings

### Having fewer family members would also cut down on communication costs in making phone calls to remind people to attend psychoeducation sessions

“It is hard enough to successfully contact one caregiver. It is almost impossible to contact many family members when they may not even be staying in the same location at the time of contacting them. They may also not have means for communication.”

Psychiatric Nurse 1

It is well documented that the cost of psychoeducation has hindered its availability even in high income countries [24]. In this regard, the nurses were against a whole day workshop which would imply providing lunch and snacks at break time for the participants. This in turn would not be financially feasible and sustainable for the current study but also for future practice.

e) Low literacy as a challenge

The nurses stated that through observations, literacy levels were low, and the postpartum women could refuse participation when they learnt that they would have to read and write in the intervention groups.

“I think there is a need to cater for those who cannot read and write. To avoid making them feel discriminated agaisnt, we should not bring in the idea of reading and writing”

“We should explain in explicit language that even without writing, participants will be able to recall the discussion. We should also be flexible enough that if some participants wanted to write and wanted to obtain reading materials, these should be catered for.”

Psychiatric Nurse 3

f) Timekeeping

Participants and moderators would agree on a convenient time for the forthcoming psychoeducation session. However some participants were regularly not coming on time and thus inconvenienced other participants. This was especially observed when it rained in the morning. This called for flexibility on the part of the moderators/psychologists and those that were on time. Hence it was noted that at the end of each session, a reminder on the importance of timekeeping would be made.

### Theme II: Content of family psychoeducation

The sub-themes within content were: inclusion of participants’ lay perceptions on causes and treatment of mental illness; family planning education; demystifying myths about breast feeding when a postpartum woman has mental illness; potential income generating activities, and finally an emphasis on personality changes that the woman undergoes when she develops the condition, before and after the psychotic episode.

a) Inclusion of lay perceptions on causes and treatment of mental illness

It was necessary to find out what the participants perceived to be the cause and treatment of the mental illness as this often influenced patients’ help-seeking behaviour [15,16]. Previous research has documented the Ganda tribe as identifying supernatural powers as being the cause of severe mental illness [25]. Often this belief leads them to non- compliance to Western treatment for mental illness. The nurses suggested exploring the patients’ cultural perceptions about causation and treatment of mental illness. These were incorporated in the psychoeducation content.

“Many patients and their caregivers believe that mental illness is a result of witchcraft or it is a clan illness. For you to introduce Western ideas about causation and treatment, you need to understand what people think regarding the perceived causes and hence the treatments.”

Psychiatric Nurse 1

b) Inclusion of Family Planning education

Some caregivers and postpartum women believed that family planning education should be made part of the psychoeducation for postpartum women.

“Can you start teaching about family planning in these sessions? My daughter has six children and each one has a different father. Every time she breaks down, she conceives with a different man! I am not able to look after all these children… You need to teach us about family planning”

Caregiver 7

Another caregiver said:

“I also wish there was family planning. My daughter breaks down every time she delivers. Her husband then brings her and dumps her at my doorstep! Then I have to take care of her and when she improves, she runs back to her husband and conceives again.”

Caregiver 3

“The only time that I fall sick is when I deliver. I need family planning”

Postpartum woman 2

And one husband of a postpartum woman said:

“This illness came on after her delivery. Is there not something that can be done because after all we have six children! These children are enough.”

Caregiver 4

The need for family planning education and services was further supported in one of the consultative meetings with the nurse who argued that postpartum women would be able to obtain these services at the same time while undergoing the psychoeducation intervention.

c) Breastfeeding

The importance of women to continue breastfeeding even when they were mentally ill was highlighted by the participants. During the discussion, some participants reported cultural beliefs that breastfeeding a baby when one was acutely mentally ill would introduce the mental illness to the baby later on in his or her life. Others expressed fears, for example once a postpartum woman stopped breast-feeding the baby, it was important not to breastfeed the baby again because the assumption was that the breast milk would be spoilt and this would make the baby physically ill. The extracts below illustrate the beliefs:

“I have been refused to breastfeed my baby again because I have been told that my breast milk carries the mental illness and my baby may grow up to eventually develop mental illness just like me”

Postpartum woman 6

“I have been told not to breastfeed my baby again because people say that my breast milk has gone sour during the time I have been away in hospital”

Postpartum woman 2

Considering that the infants of postpartum women were missing out on the benefits of breastfeeding and the fact that Uganda is a low income country, all the participants felt that the idea of stopping breastfeeding while mentally sick should be targeted in the psychoeducation intervention. This was raised in the last consultative meeting that included the women, their caregivers, the nurses and the psychologists.

d) Income-generating activities

There was indication from the group discussion that one of the reasons why women did not comply with medical treatment was the failure to obtain money for transport to the hospital for reviews or to buy medications. Coupled with this was the fact that the majority of postpartum women could not sustain employment because of the nature of their mental illness. The postpartum women and their caregivers proposed an incorporation of income generating activities in the problem solving skills of psychoeducation to financially make them competent:

“We are sometimes not able to come back for medication because we lack the money for transportation to Butabika”

Postpartum woman 1

“It is difficult for me to think of coming back to hospital when I don’t even have food in my house”

Postpartum woman 3

e) Changes in personalities of postpartum women before and after the illness

Postpartum women were of the view that their caregivers needed to understand that when the women were irritable or when they destroyed property, it was because they were ill and not that they destroyed property intentionally. Moreover it was also important for them to dispel the culturally held belief that mental illness could not be cured or improved and that once mentally ill, one is forever “mad”.

“I think that it is important to emphasize to our caregivers that although we may behave like we understand what we are doing, we are really sick. They don’t want to think that we are sick. They continue to say that we are pretending when we destroy property and when we insult them during our relapse periods.”

Postpartum woman 2

“That is true. My relatives think the same way too. They think that I purposely intend to harm them. So when I improve, no one wants to stay with me. They are very angry at me and they insist that I destroy property and insult them intentionally”

Postpartum woman 4

Although symptoms of the illness were a part of the standard psychoeducation, it was deemed necessary to put emphasis on these because the mothers thought they were being misunderstood by their caregivers as mentioned above. Furthermore, many postpartum mothers feared being branded “forever mad” and preferred to keep their mental illness a secret, even from their husbands for fear of abandonment.

A summary of standard Family psychoeducation and the modified Family psychoeducation are shown in Table 2. The derived contents specific to postpartum women with a psychotic illness in the setting were incorporated. Whereas standard family psychoeducation is concerned with educating patients and families on epidemiology of specific mental illnesses, the nurses and psychologists in the research team opted for incorporating the cultural lay perceptions on causes of mental illness since these determine the help-seeking behaviour of the patients. Family planning education, breastfeeding and income-generating activities are not included in standard family psychoeducation but were included in the modified psychoeducation.

**Table 2:** A comparison of standard family psychoeducation for bipolar disorder with the modified family psychoeducation for postpartum mothers with a psychotic illness

**Standard family psychoeducation (SFP)**	**Modified family psychoeducation**
**1. Joining**	** 1. Joining**
Identify early warning signs	Same as in standard family psychoeducation
Explore reactions to illness	** 2. Conjoint**
Identify coping strategies, triggers	Same as in standard family psychoeducation
Review family networks	**3. Education workshop**
Investigate ways to reduce burden	Instead of a whole day workshop, this was spread into other psychoeducation sessions
**2. Conjoint**	The epidemiology and biology of the illness were left out. Instead Lay perceptions including cultural beliefs about causes and treatments were incorporated, and early warning signs and triggers were dealt with in a participatory manner
Allows family to come together as a unit before joining the multifamily group	
**3. Education workshop**	** 4. Problem solving, communication and vocational training**
Whole day workshop to address history and epidemiology, biology of illness, treatment and side effects, and family emotional reactions	This remained as in standard family psychoeducation but family planning, and breast feeding were incorporated along with specific income generating activities and the main moderators were the caregivers.
**4. Problem solving, communication and vocational training**	

## Discussion

We aimed to explore the issues that would make standard family psychoeducation culturally acceptable and relevant to a postpartum population in a Ugandan setting. We observed that the basic therapeutic elements deemed necessary for a moderator to work effectively in a cross-cultural setting in psychoeducation were applicable. The study findings indicate that in the process of adapting the family psychoeducation program, the essential elements in the basic therapeutic process of effective communication, and culturally accepted norms including language, age of moderators and same sex moderators were maintained. The importance of maintaining these basic elements of psychoeducation during adaptation, so that the programme does not lose its fidelity, cannot be overemphasized [2]. Ideas that would make the programme culturally relevant and gender sensitive in the Ugandan setting were incorporated.

Previous research findings have emphasized the notion of incorporating family members when coming up with curricula for psychoeducation in order to cater for the specific needs of particular populations [26] and many of the changes in the modified psychoeducation were raised by the caregivers.

Effective communication has been described as the basis of any psychotherapeutic encounter [21]. Similar to previous research findings, participants indicated that for family psychoeducation to be effective in the Ugandan setting, the moderators had to have good communication and counselling skills as has been indicated for standard family psychoeducation in other settings [8]. Hence the moderators for our culturally sensitive family psychoeducation programme were selected on the basis of fluency in Luganda, the language that the women were fluent in to help build rapport [11]. It was important to adapt to the Ganda cultural behavioral norms for example an accepted dress code of dresses and skirts that were long past the knees. In addition, older female moderators of similar ethnic background for psychoeducation were deemed most appropriate because this would cater for cultural and gender differences, and enhance cultural identity between the postpartum women and the moderators. There are other similar findings in other studies [11,27]. Cultural appropriateness of a programme caters for the diverse dimensions of a culture including literacy. Whereas literacy rates in the west are generally high, those in low income countries are low [14] and this was accordingly integrated in the appropriateness of the programme.

Explanatory models for causation and treatment of psychotic illness may influence whether an individual complies with treatment or not [15,16]. It was therefore decided that moderators should explore Ganda perceptions on causation of mental illness in the postpartum period so that these would be adequately addressed. This has been emphasized in the dimensions of the ecological validity model. Patients’ explanatory models may not necessarily be the same as those of the moderator and if negotiation is going to occur for an effective therapeutic intervention, then the moderator has to understand the patient’s explanatory model for causation and treatment of illness [22,28] and formulate a programme that demystifies the participants’ views on illness causation and treatment if different from the evidence-based research [12].

Family psychoeducation is a successful evidence-based practice, but it is not widely applied, even in high income countries because of its high cost [7]. The nurse and psychology participants thought that if the intervention was to be integrated in the treatment of mental health conditions in a low income country, cutting down on expenses would increase the likelihood of implementing the intervention.

Although the researchers had not intended to explore the feasibility of carrying out family psychoeducation in this setting, it consistently featured as a concern and theme in the psychologists’ and nurses’ discussions and hence it was incorporated. To increase the likelihood of implementing the programme, participants suggested that instead of many family members attending sessions, one member who was most supportive should attend the sessions to reduce travel expenses.

Income generation was an important consideration by the women and their caregivers. Uganda has an unemployment rate of about 3.5% and an underemployment of about 17%. Understandably participants were preoccupied with creating jobs for both the postpartum women and the caregivers. This is further supported by the fact that many women with psychotic illness in the postpartum could have developed a bipolar illness earlier in their adolescence or even childhood and were unable to complete formal education and hence would not compete favourably for highly skilled jobs [29].

Breastfeeding was identified as an important issue that should be targeted in the psychoeducation. Mothers were not sure whether they should continue breastfeeding their babies when they developed mental illness during the postpartum period. They perceived that when a postpartum woman developed mental illness and she continued breastfeeding, the baby would grow up to develop a similar illness because of the “contaminated breast milk”. Such notions had to be dispelled. The demystifying of myths concerning cultural beliefs has been stipulated in Bernal´s ecological validity model of the 8 dimensions [11]. Family psychoeducation entails providing information and educating the concerned parties and therefore the provision of information on breast feeding was called for because it would enhance patients’ knowledge.

### Study limitations

We used a multi-method study design in order to provide the data. There was no literature to indicate that this was the most appropriate design for this particular study. There was no gold standard procedure with which to compare and determine that the cultural adaptation carried out did not lose its fidelity and there was insufficient data available to determine that the resultant cultural adaptation was superior to using standard evidence-based family psychoeducation in Uganda. However, considering the different cultural explanatory models for mental illness, the low income setting, and the low literacy levels, it is likely that the revised version would yield better outcomes in the Ugandan population than if the standard evidence-based family psychoeducation had been implemented without changes. The perceptions and views on the comprehensiveness, acceptability and value of psychoeducation were not elicited from the participants in the programme. This indicates that although we set out to include stakeholders, their views on these matters are missing from this feasibility study.

## Conclusion

The general principles of carrying out family psychoeducation in a Ugandan setting were applicable in this adaptation study as elsewhere. It was evident that interventional programme could not be separated from the socio-cultural and economic contexts of a particular population. The modifications in standard family psychoeducation in the present study reflected the unique socio-cultural and economic reality of this population.

## Competing interests

All the authors declare that they have no competing interests in the study.

## Authors’ contributions

JN and AE conceived of the idea. JN did the data collection. All authors participated in the designing of the study and the analysis. This piece of work has been read and reread by all the authors in consultation with an individual with English as a first language and they have approved it. All authors read and approved the final manuscript.

## Pre-publication history

The pre-publication history for this paper can be accessed here:

http://www.biomedcentral.com/1471-244X/13/131/prepub
